# Place of Birth Inequalities in Dental Care Use before and after the Economic Crisis in Spain

**DOI:** 10.3390/ijerph16101691

**Published:** 2019-05-14

**Authors:** Elena Rodriguez-Alvarez, Nerea Lanborena, Luisa N. Borrell

**Affiliations:** 1 Department of Nursing I, University of the Basque Country (UPV/EHU), 48940 Leioa, Bizkaia, Spain; nerea.lamborena@ehu.eus; 2 OPIK-Research Group for Social Determinants of Health and Demographic Change, University of the Basque Country (UPV/EHU) 48940 Leioa, Bizkaia, Spain; Luisa.Borrell@sph.cuny.edu; 3 Department of Epidemiology & Biostatistics, Graduate School of Public Health & Health Policy, City University of New York, New York, NY 10027, USA; 4 Department of Surgery, Medical and Social Science. University of Alcalá, 28871 Madrid, Spain

**Keywords:** dental care, immigrants, inequalities, health survey, economic crisis

## Abstract

This study evaluates inequalities in the use of dental services according to place of birth before and after the economic crisis in Spain. A cross-sectional study was performed in adults aged 18 to 65 years in Spain. We used data from three Spanish National Health Surveys for the years 2006 (before the crisis), 2014, and 2017 (after the crisis). Log-binomial regression was used to quantify the association between place of birth and use of dental care services before and after controlling for the selected covariates. In 2006, we found a greater probability of not using dental care services in immigrants from Asia (PR: 1.36, 95% CI: 1.10–1.67) and Africa (PR: 1.16, 95% CI: 1.05–1.28) compared to the natives. For 2014, the probability of not using dental care services was greater for all immigrants relative to natives, with the greatest probability for those from Africa (PR: 1.71, 95% CI: 1.46–2.01) and Asia (PR: 1.3, 95% CI: 1.23–1.47). The associations for 2017 were weaker in magnitude than the ones observed for 2014, although stronger than for 2006. This study suggests that the economic recovery did not have the same impact for natives and immigrants regardless of regions of origin, given the observed inequalities in use of dental services.

## 1. Introduction

Oral health is an important component of populations’ health status. In fact, evidence points to the relationship between oral health and cancer mortality [1], cardiovascular and respiratory diseases [2] diabetes [3], obesity [4], premature births [5], and quality of life [6]. In addition, poor oral health follows a clear socioeconomic pattern as a result of increased exposure to risk factors among individuals with low educational attainment, income, and employment status. This pattern of inequality has been described systematically both between and within countries [7,8]. One of the factors that may contribute to explain these inequalities is access to dental care. Although inequalities in access to dental care services have been described in some countries [9,10,11,12], they are more pronounced in countries where dental care is not covered by the public system compared to those countries with at least some dental care coverage [9,12,13,14,15].

In Spain, dental care services offered by the public system to the population over 16 years of age are limited to treatments for acute dental problems, such as infectious processes in the mouth, tooth trauma, or extractions [16]. Thus, adults must pay the total cost of dental care, which is the largest private expense in health for Spanish households. This cost represents an important barrier for access, contributing to inequalities in access to dental care services among socioeconomically disadvantaged groups since 1997 [14,15,17,18,19,20] and even increasing inequalities during the economic crisis [21]. The economic crisis began in 2008 and was characterized by a decline in macroeconomic indicators, such as the gross domestic product (GDP), employment, and national income. In fact, unemployment went from 9.6% in 2008 to 27.2% in 2013 [22], along with job insecurity, salary and benefit cuts, decreasing family income (up to a 31% in the lowest income decile) and an increasing proportion of the population at risk of poverty (from 3.7% in 2008 to 22.2% in 2013) [23]. Immigrants were overrepresented among those affected by the crisis [24] despite representing only 10.7% of the Spanish population in 2013 [22]. The severity of the economic crisis and its impact on the labor market stopped the strong growth of the immigrant population observed in the previous decade. However, since 2017, Spain has had the fourth highest proportion of immigrants of any European country (13%). The majority come from Latin America (38%, mainly Ecuador, Colombia, Bolivia, and Peru), Africa (18%, mainly Morocco and Senegal), Asia (7%, mainly from China) and the rest of the European countries with a Human Development Index (HDI) that is not very high (17%, mainly Romania, Bulgaria, and Ukraine).

As immigration has increased, an important body of knowledge about social inequalities in health has been generated according to the place of birth and the migratory process, with the latter constituting a central axis of inequality [25]. In the case of access to dental care inequalities, international studies show lower access for the immigrant population despite having a greater need [19,26,27,28,29,30]. In Spain, two studies have examined these inequalities, one before [19] and another during the economic crisis [20].

These studies consistently showed lowest access for the immigrant population, especially for those from Africa and Asia. However, changes in health inequalities and in access to dental care have barely been studied after the recovery of economic indicators in 2014, and thus, the end of the economic crisis. In fact, the consequences of the economic crisis on health may take longer to be evident than those produced in the socioeconomic wellbeing of families [31]. Therefore, this study aims to examine the trend in inequalities in the use of dental services between the native and immigrant populations before and after the economic crisis, using data from the National and European Health Surveys in Spain.

## 2. Materials and Methods

### 2.1. Data Source and Study Population

A cross-sectional study was conducted among the population aged 18 to 65 years, as it is the age interval where the majority of the immigrant population falls. Data from the Spanish National Health Survey (SNHS) were used for years 2006 and 2017 and from the European Health Survey in Spain (EHSS) for 2014. These surveys are conducted by the Ministry of Health, Social Services and Equality and used the same stratified multistage sampling design to obtain representative samples of the non-institutionalized Spanish population. More detailed information on the methodology of these surveys has been described previously [32]. The analyses were based on the information collected by interviewing the individuals in selected households. The sample sizes were 29,478 and 23,089 for the SNHS in 2006 and 2017, respectively, and 22,842 for the EHSS in 2014. The years of these surveys represent the period before (2006) and after the economic crisis (2014 and 2017). The response rate was 96% in 2006, 71% in 2014, and 74% in 2017.

### 2.2. Variables

The dependent variable was the self-reporting of not having a dental visit during the 12 months preceding the survey as an indicator of preventive and treated oral health [33], and it was obtained from the answer to the question regarding the last visit to the dentist. The question used was slightly different in 2006 (How long ago did you go to the dentist, stomatologist, or dental hygienist for an exam, advice, or treatment of problems with your teeth or mouth?) than in 2014 and 2017 (“When was the last time you visited a dentist on your own behalf [that is, not while only accompanying a child, spouse, etc.]?”). Answers to this question included from “less than 3 months ago”, “between 3 months and 1 year ago, “1 year or more” and “never” for 2006 and “less than 3 months” and “between 3 months and 6 months ago”, “more than 6 months but less than 12” “1 year or more” and “never” for 2014 and 2017. These answers were further categorized as (1) within a year or less, and (2) more than a year or never. Hereafter, these categories would be referred to as using or not using dental services in the past year.

The independent variable was region of origin, categorized as natives for those born in Spain and immigrants for those born in a country with not very high HDI and further categorized according to the region of origin in Europe (non-European Union, Bulgaria, and Romania), Latin America, Africa and Asia (hereafter, region or origin would be referred as place of birth). In addition, the survey year was considered as a modifying variable and categorized before the economic crisis (2006) and after the economic crisis (2014 and 2017).

Consistent with other studies [19,20], sex, age (as a continuous variable), educational attainment (primary, secondary, and university), marital or partner situation (couple or other), and labor situation (working, unemployment, and others) were included as covariates. In addition, social class was used as an indicator of socioeconomic position, based on the classification of the Spanish Society of Epidemiology [34]. Social class was assigned according to the current or most recent occupation of the respondent and categorized as Class I and II (managers, intermediate technicians and managers), Class III (qualified non-manual workers) and Class IV and V (manual workers). Finally, for self-perceived oral health problems, the surveys used the question “What is the status of your teeth?”, followed by statements “you have dental caries”, “your gums bleed during brushing or spontaneously”, and “you have missing teeth replaced by prosthesis”. Survey participants were provided with the following choices: “Yes”, “no”, “don’t know”, or “refuse to answer”. These variables were specified as yes and no.

Of the participants in the ENSE 2006 (*n* = 29,478), we excluded participants under 18 (*n* = 452) or over 65 (*n* = 7479); those born in countries with an HDI very high in 2014 (>0.80) (*n* = 624); and those with missing information on education (*n* = 104), social class (*n* = 311), employment status (*n* = 8), living arrangement (*n* = 17), or self-perceived oral health problems (*n* = 239). These exclusions resulted in an analytical sample of 20,244. Similarly, for the EHSS 2014 (*n* = 22,842), we excluded those under 18 (*n* = 521) and those over 65 (*n* = 6169); those born in countries with an HDI very high in 2014 (*n* = 375); those without information on social class (*n* = 277), living arrangement (*n* = 16), or self-perceived oral health problems (*n* = 392). These exclusions resulted in an analytical sample of 15,092. Finally, for the ENSE 2017 (*n* = 23,089), we excluded those under 18 years of age (*n* = 578) and over 65 (*n* = 6689); those born in countries with an HDI very high in 2014 (*n* = 386); and those without information on social class (*n* = 260), living arrangement (*n* = 24) or self-perceived oral health problems (*n* = 268). These exclusions resulted in an analytical sample of 14,884. Thus, an analytical sample of 50,220 was used for these analyses.

### 2.3. Statistical Analysis

Descriptive statistics were calculated for the entire population and according to place of birth for all the covariates in each survey year. In addition, the prevalence of use of dental services in the last year or more was estimated for each covariate, according to place of birth. Chi-squared and Cochran–Mantel–Haenszel tests were used to determine differences between each covariate and place of birth and between each covariate and use of dental services according to place of birth. In addition, *t*-test was used to evaluate differences in age according to use of dental services in the past year and place of birth. Log-binomial regression was used to quantify the association between place of birth and use of dental services (yes vs. no) before and after controlling for selected covariates. To determine if this association varies with the economic crisis, an interaction between survey year and place of birth was tested in the final model.

Data management procedures were conducted using SPSS 24.0. (IBM, Armonk, NY, USA), whereas the statistical analyses were conducted using SUDAAN 11.0.1 (RTI, Research Triangle Park, NC, USA) to account for the complex sampling design and yield unbiased standard error estimates. Sample sizes presented in Table 1 were unweighted, while all other estimates (proportions, standard errors, prevalence ratios (PR) and their 95% confidence intervals (95% CI)) were weighted.

## 3. Results

Table 1 shows the distribution of sociodemographic characteristics, oral health status, and use of dental services for natives and the different immigrant groups according to survey year (2006, 2014, and 2017). When compared with natives, immigrants were younger and more likely to be women (with the exception of those from Africa), less educated, be in the lowest social class and be unemployed (except among those from Asia). Immigrants were less likely to have used dental services in the past year (except among those from Europe and Latin America in 2006) and more likely to report oral health problems. It is worth noting that there was little difference in the proportion of unemployment between natives and immigrants, with the exception of immigrants from Asia in 2006. However, in 2014 and 2017, unemployment was 50% higher in immigrants than in natives. Likewise, the difference in the proportion of not using dental services in the past year between natives and immigrants increased in 2014 and 2017 compared to 2006, with the greatest differences in those from Africa and Asia when compared to natives (all values *p* < 0.05).

Compared to natives, immigrants had a higher prevalence of not using dental services in the past year, associated with being a man, having a primary education and being in the most disadvantaged social class (Table 2). This pattern was consistent for all immigrant groups across survey years, with some exceptions. Being a woman was associated with a higher prevalence of not using dental services in the past year among Asian immigrants (2006 and 2014), while for Latin America and Europe, the prevalence estimates were lower than in the natives for the less educated and the most disadvantaged class. With regards to employment, unemployed immigrants from Europe and Latin America had a greater prevalence of not using dental services in the past year (except in 2017 for Latin America) compared with natives. In contrast, employed immigrants from Africa (except in 2017) and Asia had a higher prevalence of not having used dental services in the past year relative to natives. Finally, and compared with natives, immigrants generally had a higher prevalence of not using dental services in the past year associated with self-perceived oral health problems (all values *p* < 0.01).

Heterogeneity for the association between place of birth and dental care was observed during the economic crisis (*p*-interaction of place of birth and survey year = 0.0364). The latter underscored the effect of the crisis and suggested that immigrants not only differ in dental care use but also across survey years. Table 3 shows the prevalence ratios and corresponding 95% CIs for place of birth for use of dental health services according to survey year. We observed similar associations in the unadjusted and adjusted estimates across the three surveys with few exceptions. Consistent with the unadjusted analysis, in 2006, the adjusted probability of not using dental services in the past year was higher among immigrants from Asia (PR: 1.36, 95% CI: 1.10–1.67) and Africa (PR: 1.16, 95% CI: 1.05–1.28) compared with natives. This higher probability was observed for all immigrants regardless of place of birth in 2014, with the greatest probabilities in immigrants from Asia (PR: 1.71, 95% CI: 1.46–2.01) and Africa (PR: 1.34, 95% CI: 1.23–1.47). In 2017, the probabilities of not having used dental services in the past year remained significant for all immigrants relative to natives. However, relative to 2014, there was a reduction for the estimates among immigrants from Asia (PR: 1.44, 95% CI: 1.22–1.69), Africa (PR: 1.26, 95% CI: 1.15–1.39), and Latin America (PR: 1.10 95% CI: 1.01–1.20). In contrast, this association increased among immigrants from Europe (PR: 1.25, 95% CI: 1.11–1.39).

## 4. Discussion

Our findings show a greater probability of not using dental services in immigrants compared to natives, a probability that remained nearly identical after adjusting for selected characteristics including socioeconomic indicators. This association varies with survey year. While there was an association for each year and strongest for immigrants from Asia and Africa, the probability of not using dental services in the past year was greater in 2014 (or the year when the economy started the recovery) than in 2006 and 2017. The association for 2017, although higher than for 2006, was smaller in magnitude than the one observed for 2014.

In Spain, during the economic crisis and with the austerity policies implemented in the health services, the proportion of the population with unmet medical needs increased, including dental needs [35]. This increase in unmet needs due to the cost of services occurred especially in the lowest income quintiles. Although there are few studies accounting for place of birth on the inequalities in the use of dental services, and even fewer including some of the immigrant groups considered in our study, their findings are consistent with ours, showing lower use of dental services in immigrants from Africa and Asia [19,29,36,37]. These studies suggested lack of dental insurance, the cost of services, and lower income as possible explanations for these inequalities.

Our findings showed that the inequalities in dental care use were greater after the start of the economic recovery (2014) for all immigrants, especially for those from Africa and Asia. Although there is little evidence on the long-term effect of the crisis on access to dental services, a study conducted in the United States [38] had examined changes in the use of health services, including dental services, before, during, and after an economic crisis. As in our study, this study found that the utilization of dental services in the past year decreased before and during the economic crisis and continued to increase after it ended. In addition, this decrease in use of dental services was greater among immigrants compared to the natives.

Reasons related to the consequences of the years of economic crisis can help explain the increase in these inequalities between immigrant and native populations, especially in some immigrant groups in 2014. For instance, at the beginning of the economic recovery, immigrants, specifically those from Africa, were already starting from more vulnerable economic positions, since they were overrepresented in the labor sectors most affected by the economic recession, such as construction [24]. Thus, unemployment and loss of incomes together with less protection of social security and more fragile family networks may have left immigrants in a situation of greater economic deprivation and instability [24,39]. Moreover, given the loss of income together with the fact that dental services are not covered by the public health system in Spain, requiring out-of-pocket payment, it is possible that the use of dental services may not be considered a priority. In fact, previous studies show that when in the presence of loss of income, people tend to restrict the use of health services that are considered less essential, such as preventive services [40], among which dental services may be included [9,21,38]. Finally, the increase in precarious work conditions and the progressive decrease of wages that has taken place in Spain after the Labor Reform of 2012 may explain the persistence of inequalities in the use of dental health services after three years of economic growth and employment growth [18,41]. In fact, after the labor reform, social and income inequalities have increased and, as a consequence, the access to goods and services has decreased [35]. Thus, the availability of economic resources may have a more relevant role than the employment situation for the use of dental services and the increase of inequalities between immigrant and native populations [42].

This study has some limitations that must be considered. The study is based on self-reported information, which can bias the results on either direction, over or underestimation. However, if bias occurred, it is possible that it was non-differential, and thus, biases our estimates toward the null. In addition, when dealing with population surveys, there is a lower participation of the immigrant population with lower educational attainment, social position, and knowledge of the language. The latter may be underestimating the inequalities between natives and immigrants [43]. Moreover, information on length of residence in Spain was unavailable for the immigrant population for the 2006 survey, and there were many missing values for the 2014 and 2017 surveys. Therefore, we could not assess the role of length of residence during the analyses. However, if length of residence were to be related to dental needs in immigrants, it is possible that access to such information may have masked the true inequalities between immigrant and native populations in our study. In addition, the sample size, especially among immigrants from Asia, limits the inferences of our results. However, the disaggregation by regions of origin is already an important advance with respect to most of the studies that have presented the entire immigrant population as a homogenous group [27,28,42]. Despite these limitations, to the best of our knowledge, this study represents the first to examine inequalities in the use of dental services between natives and immigrants according to place of birth. In addition, this study uses representative samples of the Spanish population from three National Health Surveys, one before the economic crisis (2006) and two after the economic crisis (2014 and 2017). Finally, given the study’s large size, it was possible to control for relevant covariates and examine interactions.

## 5. Conclusions

Our findings show that the economic recovery has not had the same impact for natives and immigrants, given the observed inequalities in access to dental services and their increase among natives and immigrants regardless of regions of origin. Henceforth, it is necessary to continue monitoring and examining the long-term consequences of the economic crisis, given that the socioeconomic situation in Spain is expected to take time to recover to the levels it was prior to the economic crisis. The inclusion of dental health services in the portfolio of public health services is considered necessary to reduce the consequences of the crisis in the inequalities in oral health and as an important component of the health status of the population. This measure is key to facilitating access to dental health services for the most disadvantaged groups with greater restrictions on their access, such as immigrants, to achieve health equity.

## Figures and Tables

**Table 1:** Distribution of selected characteristics for participants of the Spanish National Health Survey 2006 and 2017 and European Health Survey in Spain 2014 according to place of birth.

	2006						2014						2017					
Characteristics	Natives *n* = 18,682 % (SE)	Europe *n* = 306 % (SE)	Latin America *n* = 843 % (SE)	Africa *n* = 366 % (SE)	Asia *n* = 47 % (SE)		Natives *n* = 13,800 % (SE)	Europe *n* = 264 % (SE)	Latin America *n* = 658 % (SE)	Africa *n* = 320 % (SE)	Asia *n* = 50 % (SE)		Natives *n* = 13,346 % (SE)	Europe *n* = 315 % (SE)	Latin America *n* = 736 % (SE)	Africa *n* = 402 % (SE)	Asia *n* = 85 % (SE)	
**Age (years)**	40.9 (0.1)	31.5 (0.7)	35.3 (0.5)	34.1 (0.8)	38.9 (2.4)	*	42.7 (0.1)	36.8 (0.8)	37.7 (0.6)	38.2 (0.9)	37.3 (1.9)	*	43.4 (0.1)	38.8 (0.7)	38.9 (0.5)	39.2 (0.6)	36.0 (1.4)	*
**Gender**						*						*						
Men	51.1 (0.5)	49.7 (3.9)	40.2 (2.4)	64.8 (3.6)	44.2 (14.5)		50.8 (0.5)	45.3 (3.7)	41.3 (2.4)	52.7 (3.9)	38.3 (7.8)		50.9 (0.5)	43.8 (3.2)	38.1 (2.1)	52.6 (3.0)	55.7 (6.1)	
Women	48.9 (0.5)	50.3 (3.9)	59.8 (2.4)	35.2 (3.6)	55.8 (14.5)		49.2 (0.5)	54.7 (3.7)	58.7 (2.4)	47.3 (3.9)	61.7 (7.8)		49.1 (0.5)	56.2 (3.2)	61.9 (2.1)	47.4 (3.0)	44.3 (6.1)	
**Educational attainment**						*						*						*
Primary or less	47.3 (0.5)	33.1 (3.6)	37.9 (2.3)	69.8 (3.5)	35.9 (9.8)		43.6 (0.5)	41.0 (3.8)	37.6 (2.4)	77.5 (3.0)	39.5 (7.8)		41.9 (0.5)	31.7 (3.1)	33.9 (2.0)	70.9 (2.7)	48.0 (6.1)	
Secondary	32.2 (0.5)	53.6 (3.9)	43.4 (2.4)	22.8 (3.2)	49.9 (9.8)		33.1 (0.5)	45.8 (3.7)	44.6 (2.4)	15.6 (2.7)	32.5 (7.5)		34.4 (0.5)	55.8 (3.2)	47.8 (2.1)	21.9 (2.5)	29.3 (5.6)	
Graduate or higher	20.5 (0.4)	13.3 (2.6)	18.7 (1.9)	7.4 (1.8)	14.2 (5.9)		23.3 (0.4)	13.2 (2.5)	17.7 (1.6)	6.9 (1.5)	28.0 (7.7)		23.7 (0.4)	12.5 (2.1)	18.3 (1.6)	7.2 (1.5)	22.7 (5.1)	
**Social Class**						*						*						*
I–II	22.6 (0.4)	6.1 (1.7)	13.2 (1.6)	5.9 (1.8)	17.9 (8.0)		22.1 (0.4)	5.2 (1.8)	10.5 (1.3)	2.2 (0.8)	12.7 (5.2)		22.0 (0.4)	6.3 (1.7)	11.5 (1.4)	2.8 (0.9)	11.5 (3.9)	
III	25.4 (0.4)	9.2 (2.5)	10.5 (1.6)	8.2 (2.1)	14.0 (5.5)		20.8 (0.4)	8.1 (2.2)	11.2 (1.4)	6.3 (1.6)	47.7 (8.2)		20.6 (0.4)	7.5 (1.7)	12.8 (1.4)	8.1 (1.7)	23.2 (5.3)	
IV–V	52.0 (0.5)	84.7 (2.9)	76.3 (2.1)	85.8 (2.7)	68.1 (8.9)		57.1 (0.5)	86.7 (2.7)	78.3 (1.8)	91.5 (1.8)	39.5 (7.9)		57.4 (0.5)	86.2 (2.3)	75.6 (1.8)	89.1 (1.9)	65.3 (5.9)	
**Employment status**						*												*
Employed	63.2 (0.5)	79.8 (3.0)	78.0 (1.9)	62.4 (3.8)	65.3 (9.4)		58.9 (0.5)	59.3 (3.7)	58.6 (2.4	33.7 (3.4)	62.5 (7.9		62.6 (0.5	64.2 (3.1)	64.4 (2.0)	53.1 (3.0)	57.9 (6.1)	
Unemployed	8.8 (0.3)	9.2 (2.1)	9.0 (1.4)	9.4 (2.4)	14.7 (8.2)		18.5 (0.4)	27.2 (3.2)	25.3 (2.1)	37.9 (3.8)	3.7 (2.5)		14.9 (0.4)	21.6 (2.6)	21.0 (1.7)	20.0 (2.3)	12.2 (3.9)	
Others	28.0 (0.4)	11.0 (2.3)	13.0 (1.6)	28.2 (3.6)	19.9 (6.8)		22.6 (0.4)	13.5 (2.7)	16.1 (1.9)	28.4 (3.6)	33.8 (7.8)		22.5 (0.4)	14.2 (2.3)	14.6 (1.5)	26.9 (2.7)	26.9 (5.9)	
**Living arrangement**						*						*						*
Married/Couple	59.5 (0.5)	52.4 (3.9)	48.7 (2.4)	61.5 (4.0)	78.0 (8.4)		59.9 (0.5)	65.8 (3.5)	54.6 (2.4)	71.0 (3.6)	74.6 (7.3)		61.2 (14.5)	65.0 (3.1)	54.7 (2.1)	72.0 (2.7)	61.6 (6.0)	
Other	40.5 (0.5)	47.6 (3.9)	51.3 (2.4)	38.5 (4.0)	22.0 (8.4)		40.1 (0.5)	34.2 (3.5)	45.4 (2.4)	29.0 (3.6)	25.4 (7.3)		38.8 (14.5)	35.0 (3.1)	45.3 (2.1	28.0 (2.7)	38.5 (6.0)	
**Use of dental care**																		*
<1 year	41.4 (0.5)	42.8 (3.9)	40.9 (2.3)	27.8 (3.3)	21.8 (7.8)	*	50.8 (0.5)	39.6 (3.7)	43.8 (2.4)	24.7 (3.3)	21.7 (5.9)	*	55.1 (0.5)	42.1 (3.2)	51.5 (2.1)	35.5 (2.9)	35.5 (5.9)	
≥1 year/never	58.6 (0.5)	57.2 (3.9)	59.1 (2.3)	72.2 (3.3)	78.2 (7.8)		49.2 (0.5)	60.4 (3.7)	56.2 (2.4)	75.3 (3.3)	78.3 (5.9)		44.9 (0.5)	57.9 (3.2)	48.5 (2.1)	64.5 (2.9)	64.5 (5.9)	
**Self-perceived oral health problems**																		
Dental caries	30.4 (0.5)	58.6 (3.8)	32.6 (2.3)	31.0 (3.5)	28.0 (8.9)	*	26.3 (0.5)	39.1 (3.6)	34.5 (2.3)	38.8 (3.7)	31.7 (7.9)	*	22.6 (0.4)	36.5 (3.1)	25.5 (1.9)	36.2 (2.9)	18.4 (5.0)	*
Lost teeth	50.8 (0.5)	56.4 (3.9)	48.9 (2.4)	41.8 (3.8)	37.5 (10.1)	**	54.6 (0.5)	58.1 (3.8)	50.0 (2.4)	56.9 (3.8)	39.3 (8.0)		58.5 (0.5)	65.6 (3.1)	58.6 (2.1)	62.4 (2.9)	36.8 (5.8)	*
Gingival bleeding	24.1 (0.4)	27.3 (3.7)	27.0 (2.2)	26.8 (3.5)	13.1 (6.3)		19.4 (0.4)	19.5 (2.8)	20.0 (1.8)	23.7 (3.2)	9.6 (4.0)		19.0 (0.4)	19.3 (2.5)	17.9 (1.7)	18.6 (2.4)	12.5 (4.2)	

*p*-values from Chi-square statistics and *t*-tests. * *p* < 0.01 ** *p* < 0.05.

**Table 2:** Prevalence estimates for non-use of dental care in the last year according to place of birth by year of the survey: Spanish National Health Survey 2006 and 2017 and European Health Survey in Spain 2014.

	2006						2014						2017					
Characteristics	Natives *n* = 18,682 % (SE)	Europe *n* = 306 % (SE)	Latin America *n* = 843 % (SE)	Africa *n* = 366 % (SE)	Asia *n* = 47 % (SE)		Natives *n* = 13,800 % (SE)	Europe *n* = 264 % (SE)	Latin America *n* = 658 % (SE)	Africa *n* = 320 % (SE)	Asia *n* = 50 % (SE)		Natives *n* = 13,346 % (SE)	Europe *n* = 315 % (SE)	Latin America *n* = 736 % (SE)	Africa *n* = 402 % (SE)	Asia *n* = 85 % (SE)	
**Overall Prevalence**	56.6 (0.5)	57.2 (3.9)	59.2 (2.3)	72.2 (3.3)	78.2 (7.8)	*	49.2 (0.5)	60.4 (3.7)	56.2 (2.4)	75.3 (3.3)	78.3 (5.9)	*	44.9 (0.5)	57.9 (3.2)	48.5 (2.1)	64.5 (2.9)	64.5 (5.9)	*
**Gender**						*						*						
Men	62.5 (0.7)	60.4 (5.9)	64.8 (3.8)	76.3 (4.0)	68.7 (14.5)		53.6 (0.7)	67.5 (5.3)	62.5 (3.7)	80.0 (4.0)	75.8 (10.3)		48.9 (0.7)	62.8 (4.8)	56.4 (3.4)	72.4 (3.8)	73.8 (6.9)	
Women	54.7 (0.6)	50.3 (3.9)	55.3 (2.9)	64.7 (5.7)	85.7 (7.4)		44.7 (0.7)	54.5 (5.0)	51.7 (3.0)	70.0 (5.4)	79.9 (7.2)		40.7 (0.7)	54.1 (4.2)	43.7 (2.7)	55.7 (4.4)	53.0 (9.6)	
**Educational attainment**						*						*						*
Primary or less	64.2 (0.7)	60.8 (6.4)	62.8 (3.7)	73.1 (4.1)	88.0 (8.8)		57.8 (0.8)	70.6 (6.0)	67.5 (3.8)	76.9 (3.8)	92.3 (4.9)		53.6 (0.8)	64.6 (5.7)	55.0 (3.7)	66.6 (3.4)	79.7 (7.2)	
Secondary	51.1 (0.9)	58.5 (15.5)	59.3 (3.5)	68.5 (6.9)	66.9 (13.2)		45.2 (0.9)	52.6 (5.2)	52.2 (3.6)	72.6 (8.6)	75.1 (10.7)		41.7 (0.9)	56.8 (4.2)	49.3 (3.1)	58.7 (6.4)	52.7 (11.3)	
Graduate or higher	51.3 (1.1)	43.3 (10.2)	51.5 (5.5)	74.9 (10.4)	93.2 (5.7)		38.9 (1.0)	55.6 (9.7)	42.3 (4.9)	63.4 (10.6)	62.3 (15.3)		34.0 (1.0)	45.7 (9.0)	34.6 (4.5)	61.4 (10.5)	47.8 (12.8)	
**Social Class**						*						*						*
I–II	51.5 (1.1)	37.8 (14.2)	57.3 (6.4)	64.2 (13.6)	98.4 (1.7)		39.6 (1.1)	45.1 (18.0)	42.4 (6.3)	54.5 (17.4)	49.0 (22.0)		35.8 (1.1)	40.5 (14.0)	33.4 (5.8)	71.9 (14.5)	31.0 (17.9)	
III	57.3 (1.0)	63.3 (12.9)	49.0 (8.1)	75.2 (10.1)	61.5 (20.4)		44.1 (1.1)	30.1 (11.1)	38.7 (6.4)	61.9 (12.6)	87.2 (6.7)		39.4 (1.1)	56.8 (11.9)	36.2 (5.7)	28.1 (8.9)	59.5 (13.0)	
IV–V	62.4 (0.7)	58.0 (4.2)	60.9 (2.6)	72.5 (3.6)	76.3 (10.2)		54.8 (0.7))	64.2 (3.8)	60.5 (2.7)	76.7 (3.5)	77.0 (9.1)		50.3 (1.1)	59.3 (3.4)	53.0 (2.4)	67.6 (3.0)	72.2 (6.8)	
**Employment status**						*												*
Employed	57.3 (0.6)	57.0 (4.5)	59.2 (2.6)	74.3 (3.9)	81.5 (9.8)		45.3 (0.6)	56.9 (4.9)	55.0 (3.0)	81.4 (4.3)	81.3 (7.0)		41.0 (0.6)	58.2 (3.9)	49.1 (2.7)	67.4 (3.9)	67.4 (7.3)	
Unemployed	63.3 (1.7)	72.4 (10.0)	70.2 (6.6)	73.9 (10.9)	63.4 (26.5)		57.1 (1.2)	69.3 (5.9)	60.2 (4.6)	70.4 (5.9)	54.0 (34.6)		54.3 (1.4)	57.5 (6.9)	49.7 (4.6)	73.7 (5.5)	63.2 (16.3)	
Others	60.1 (0.9)	46.5 (11.2)	51.3 (6.4)	67.1 (7.0)	78.4 (14.3)		52.9 (1.1)	57.7 (10.9)	54.3 (6.6)	74.5 (6.8)	75.4 (11.0)		49.4 (1.2)	57.1 (8.9)	44.6 (5.5)	52.0 (6.0)	59.5 (12.0)	
**Living arrangement**						*						*						*
Married/Couple	58.6 (0.6)	56.8 (5.4)	56.6 (3.3)	69.5 (4.1)	76.7 (9.3)		49.1 (0.6)	63.3 (4.5)	52.5 (3.2)	70.7 (4.2)	73.9 (7.5)		44.7 (0.6)	59.3 (3.9)	47.1 (2.9)	64.9 (3.4)	72.0 (7.1)	
Other	58.7 (0.8)	57.7 (5.7)	61.6 (3.3)	76.6 (5.5)	83.4 (12.8)		49.4 (0.9)	54.8 (6.3)	60.6 (3.6)	86.4 (4.2)	91.3 (7.1)		45.2 (0.8)	55.3 (5.4)	50.3 (3.1)	63.5 (5.6)	52.6 (9.9)	
**Self-perceived oral health problems**																		
Dental caries	65.7 (0.9)	61.1 (5.1)	70.4 (3.9)	63.1 (6.4)	82.0 (11.5)	*	60.2 (1.0)	68.0 (4.9)	66.8 (4.0)	73.9 (5.2)	73.8 (12.6)		60.8 (1.1)	58.7 (5.4)	56.8 (4.4)	58.6 (4.9)	90.0 (6.6)	*
Lost teeth	59.3 (0.7)	50.2 (5.2)	57.4 (3.3)	67.2 (5.0)	76.2 (14.1)	*	49.3 (0.7)	58.9 (4.6)	56.2 (3.3)	71.0 (4.5)	76.7 (9.8)		45.9 (0.7)	56.5 (3.9)	50.5 (2.8)	65.2 (3.6)	65.8 (9.4)	*
Gingival bleeding	61.0 (1.0)	57.1 (8.2)	60.3 (4.7)	65.1 (7.2)	27.5 (21.8)		52.7 (1.1)	56.9 (8.1)	62.3 (4.8)	71.4 (7.4)	94.3 (6.0)	*	49.4 (1.2)	52.5 (7.3)	46.9 (5.2)	42.0 (7.1)	55.4 (17.8)	*

*p*-values from Cochran–Mantel–Haenszel statistics. * *p* < 0.01.

**Table 3:** Prevalence Ratios and their 95% confidence intervals of non-use of dental care in the last year for place of birth by year of the survey: Spanish National Health 2006 and 2017 and European Health Survey in Spain 2014.

	Unadjusted PR (95% CI)	Adjusted * PR (95% CI)
2006		
Natives Europe Africa Latin America Asia	1.00 0.97 (0.85–1.12) 1.23 (1.12–1.35) 1.01 (0.94–1.10) 1.33 (1.10–1.62)	1.00 0.95 (0.83–1.10) 1.16 (1.05–1.28) 1.03 (0.96–1.12) 1.36 (1.10–1.67)
2014		
Natives Europe Africa Latin America Asia	1.00 1.23 (1.09–1.38) 1.53 (1.40–1.67) 1.14 (1.05–1.24) 1.59 (1.37–1.85)	1.00 1.20 (1.07–1.35) 1.34 (1.23–1.47) 1.13 (1.05–1.23) 1.71 (1.46–2.01)
2017		
Natives Europe Africa Latin America Asia	1.00 1.29 (1.16–1.44) 1.44 (1.31–1.57) 1.08 (0.99–1.18) 1.44 (1.20–1.72)	1.00 1.25 (1.11–1.39) 1.26 (1.15–1.39) 1.10 (1.01–1.20) 1.44 (1.22–1.69)

* Adjusted for age, sex, self-perceived oral health problems (dental caries, gingival bleeding and missing teeth), employment status, living arrangement, social class, and education attainment.
